# Phosphomannomutase 2 (PMM2) variants leading to hyperinsulinism-polycystic kidney disease are associated with early-onset inflammatory bowel disease and gastric antral foveolar hyperplasia

**DOI:** 10.1007/s00439-023-02523-7

**Published:** 2023-02-11

**Authors:** Fevronia Kiparissi, Antonia Dastamani, Liina Palm, Aline Azabdaftari, Luis Campos, Edward Gaynor, Stephanie Grünewald, Holm H. Uhlig, Robert Kleta, Detlef Böckenhauer, Kelsey D. J. Jones

**Affiliations:** 1 Department of Paediatric Gastroenterology & Nutrition, Great Ormond Street Hospital for Children NHS Foundation Trust, London, UK; 2 Department of Paediatric Endocrinology, Great Ormond Street Hospital for Children NHS Foundation Trust, London, UK; 3 Department of Histopathology, Great Ormond Street Hospital for Children NHS Foundation Trust, London, UK; 4 Translational Gastroenterology Unit, Nuffield Department of Clinical Medicine, University of Oxford, Oxford, UK; 5 Department of Metabolic Medicine, Great Ormond Street Hospital for Children NHS Foundation Trust, London, UK; 6 Department of Paediatrics and Biomedical Research Centre, University of Oxford, Oxford, UK; 7 Department of Renal Medicine, University College London, London, UK; 8 Department of Paediatric Nephrology, Great Ormond Street Hospital for Children NHS Foundation Trust, London, UK; 9 The Kennedy Institute of Rheumatology, Nuffield Department of Orthopaedics, Rheumatology, and Musculoskeletal Sciences, University of Oxford, Oxford, UK

## Abstract

Phosphomannomutase 2 (PMM2) deficiency causes Congenital Disorder of Glycosylation (PMM2-CDG), but does not have a recognised association with Inflammatory Bowel Disease (IBD). A distinct clinical syndrome of hyperinsulinism and autosomal recessive polycystic kidney disease (HIPKD) arises in the context of a specific variant in the *PMM2* promotor, either in homozygosity, or compound heterozygous with a deleterious *PMM2* variant. Here, we describe the development of IBD in three patients with PMM2-HIPKD, with onset of IBD at 0, 6, and 10 years of age. In each case, intestinal inflammation coincided with the unusual finding of gastric antral foveolar hyperplasia. IBD disease was of variable severity at onset but well controlled with conventional and first-line biologic treatment approaches. The organ-level pattern of disease manifestations in PMM2-HIPKD-IBD may reflect a loss of *cis*-acting regulatory control by hepatocyte nuclear factor 4 alpha (HNF4A). Analysis of published transcriptomic data suggests that IBD most likely arises due to an impact on epithelial cellular function. We identify a specific pattern of variation in *PMM2* as a novel association of early-onset IBD with distinctive gastric pathology.

**Supplementary Information:**

The online version contains supplementary material available at 10.1007/s00439-023-02523-7.

## Introduction

Genetic variation contributes substantially to the multifactorial aetiopathogenesis of Inflammatory Bowel Disease (IBD) (Graham and Xavier 2020). While this contribution is generally polygenic, in some patients, IBD-like inflammation arises consequent to a highly penetrant monogenic disorder (Ouahed et al. 2020). Identification of monogenic drivers of IBD can facilitate the implementation of specific personalised treatment strategies, and has contributed to our understanding of the biology of induction and resolution of inflammation in the gastrointestinal tract (Uhlig and Powrie 2018).

Phosphomannomutase 2, encoded by the *PMM2* gene, is a cytosolic enzyme catalysing one of the first steps of the N-glycosylation pathway, which is responsible for the post-translational modification of a diverse array of proteins and lipids. Biallelic deleterious variants in *PMM2* underlie the commonest Congenital Disorder of Glycosylation (CDG) disease (PMM2-CDG). PMM2-CDG is a complex multisystem disorder. Although diarrhoea is relatively common, sometimes associated with minor, focal enteropathy (Schiff et al. 2017; Altassan et al. 2019), there is no recognised association with IBD-like intestinal inflammation (Schiff et al. 2017; Francisco et al. 2020). We recently reported a cohort of patients affected by hyperinsulinaemic hypoglycaemia (HI) and autosomal recessive polycystic kidney disease (HIPKD) and identified a specific underlying variant in the promotor of *PMM2*, which was found either in homozygosity or in *trans* with deleterious variants in *PMM2* (Cabezas et al. 2017). Here, we report that three of these patients have additionally developed Inflammatory Bowel Disease (IBD) in childhood, and manifest a distinctive pattern of gastric antral disease involvement.

## Case series

**Fig. 1:**
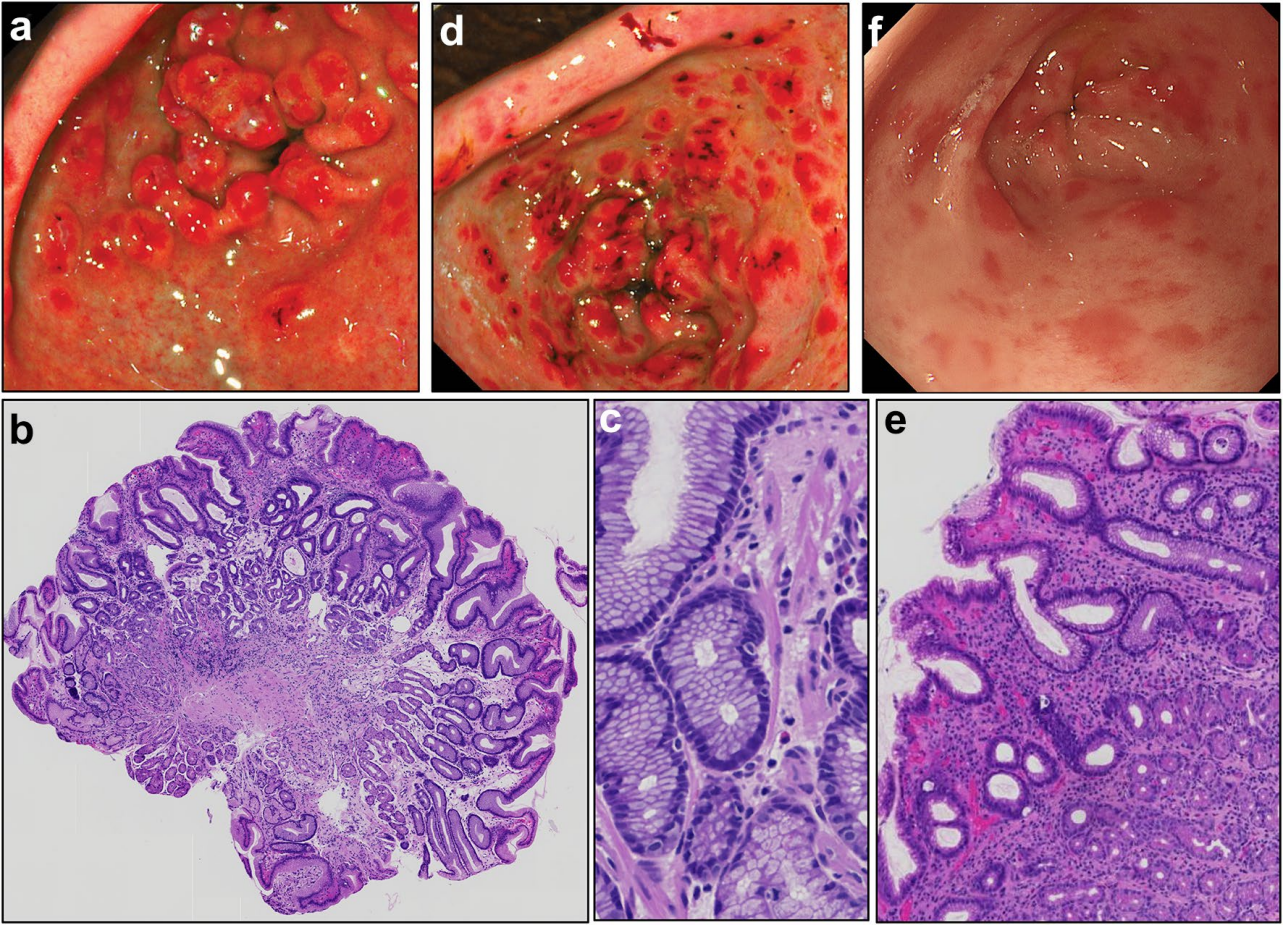
**a** Endoscopic view of the gastric antrum for P1 demonstrating a polypoid appearance at the pylorus with minor visible surface erosion. **b, c** Representative gastric antral polypoid pathology from P1 demonstrating a polypoid mucosal appearance with elongated and tortuous, corkscrew-like foveolae, and **c** smooth muscle wisps extending to the surface. **d** Endoscopic view of the gastric antrum for P2 demonstrating mucosal surface irregularity, patchy redness, and the suggestion of a minor degree of polypoid change at the pylorus with more significant surface erosions and sub-mucosal haemorrhage. **e** Representative gastric antral pathology from P2 demonstrating focal elongation, branching, and dilatation of the gastric pits with additional moderately chronic active inflammatory changes. **f** Endoscopic view of the gastric antrum for P3 demonstrating patchy redness

Patient 1 (P1, male, European) presented at 6 months of age with bloody diarrhoea and eczema, unresponsive to dietary dairy restriction. At endoscopy, there were macroscopic features of inflammation in the oesophagus, stomach, duodenum, and throughout the colon. The presence of ‘gastric polyps’ was noted, but polypectomy was not attempted due to the age of the patient, and a pictorial record was not preserved. Histopathologically, there were minor inflammatory changes in the oesophagus and stomach, and chronic inflammation with villous blunting and crypt hyperplasia in the duodenum (Supplementary Fig. 1a). In the colon, there was moderate chronic active pancolitis with architectural distortion, cryptitis, crypt abscess formation, and Paneth cell metaplasia (Supplementary Fig. 1b, c). There were no granulomata. A course of oral corticosteroids was associated with remission and P1 was maintained on azathioprine and sulfasalazine. At 13 months of age, symptomatic hypoglycaemic episodes were identified in the context of fasting. Hyperinsulinism (HI) was confirmed, and he commenced treatment with diazoxide and chlorothiazide. At 17 months of age, persistent asymptomatic hypertension led to the identification of polycystic kidney disease (PKD), and treatment with enalapril, spironolactone, and furosemide was initiated. Gastrointestinal symptoms abated over the following years and azathioprine, then sulfasalazine, were stopped. However, despite there being no upper gastrointestinal symptoms, on follow-up endoscopies gastric antral abnormalities continued to be evident, with the development of foveolar hyperplasia and even a hyperplastic polypoid appearance, which has persisted (Fig. 1a, b, c). *Helicobacter* infection was never identified, and the appearances were unresponsive to treatment with lansoprazole, to a trial of swallowed viscous budesonide, or a repeated course of azathioprine. The patient is presently 6 years of age, symptom free, thriving, with no neurological or developmental concerns, stable on treatment with respect to his hyperinsulinism and hypertension, and receives no immunomodulatory IBD-directed drugs.

Patient 2 (P2, male, European) had an antenatal diagnosis of PKD based on ultrasound findings. He developed symptomatic hyperinsulinaemic hypoglycaemia in the first few days of life. He was managed with diazoxide, chlorothiazide, amlodipine, and propranolol. He received a living-related renal transplant at 2 years of age as a result of deteriorating renal function and hypertension. As part of the pre-transplant workup, although asymptomatic, he underwent oesophago-gastro-duodenoscopy (OGD) specifically to rule out the presence of varices in view of the general risk of portal hypertension in PKD, and ‘gastropathy’ was noted. At 10 years of age, he developed bloody-mucoid diarrhoea. At endoscopy, patchy gastric antral redness was noted, corresponding with foveolar hyperplasia (Fig. 1d) and active inflammation with neutrophils invading the glandular epithelium (Fig. 1e). There was moderately severe patchy inflammatory activity through the colon macroscopically, corresponding with histopathologic findings of chronic active pancolitis with cryptitis and some epithelial apoptosis, but no crypt abscesses or granulomata (Supplementary Fig. 1d). Small intestine was normal endoscopically and on MRI. P2 was commenced on azathioprine and infliximab at diagnosis in order to secure prompt disease control since a gradual decline in renal allograft function meant that a further transplant was planned. He achieved stable remission within a few weeks. Hyperinsulinism is well controlled on treatment, he is thriving, with no neurological, developmental or other concerns.

Patient 3 (P3, male, European) had HI and PKD diagnosed in the first few days of life, having developed symptomatic hypoglycaemia and hypertension. He was managed with diazoxide, chlorothiazide, and propranolol—subsequently switched to enalapril. He had eczema that was difficult to manage, and Type 1 hypersensitivity (anaphylaxis) to egg. At 6 years of age, he developed watery and mucoid diarrhoea. Endoscopy revealed eosinophilic oesophagitis (mucosal oedema, longitudinal furrows, and an eosinophilic infiltrate of 46 eosinophils per × 40 high power field with eosinophilic microabscesses). Discrete red patches were noted in the gastric antrum, corresponding to a degree of foveolar hyperplasia with non-specific chronic active gastritis (Fig, 1f). The small intestine was macroscopically normal. In the colon, there were multiple discrete shallow ulcers with intervening areas of normal tissue, predominantly in the left colon. Histopathology confirmed patchy active inflammation with cryptitis and crypt abscesses. There were no granulomata, and no eosinophilic infiltration. He has been initiated on treatment with systemic corticosteroids and azathioprine. Hyperinsulinism and hypertension are well controlled on treatment, he is thriving with no neurological or developmental concerns.

The patients were members of a cohort with HI and PKD, who have been previously reported (Cabezas et al. 2017). They all carry the promoter variant in *PMM2* (c.-167G > T) *in trans* with a pathogenic variant (c.422G > A; p.Arg141His).

Protein expression of PMM2 and HNF4A (see discussion, below) was assessed by immunohistochemistry in P1 and P2 (Supplementary Fig. 2). PMM2 staining was most prominent in the epithelium. There appeared to be reduced protein expression for P1 compared to control, especially in the gastric antrum and colon, but for P2 the expression profile closely matched the control sample.

None of the patients had recurrent or atypical infections suggesting a primary immunodeficiency, and immunologic workups (including lymphocyte subsets, IgA, IgG, IgM, IgE, Tetanus & Pneumococcal vaccine responses, neutrophil oxidative burst, functional evaluation of SAP/XIAP) were unremarkable for P1 and P2. P3 has elevated total IgE (350 KU/L) and specific IgEs to multiple food and environmental allergens. P1 and P2 have had targeted genetic analysis of a panel of monogenic IBD-associated genes, with no pathogenic variants identified. Transferrin isoelectric focusing was normal in all the patients.

## Discussion

The observation of intestinal inflammation and gastric antral foveolar hyperplasia in three patients with identical pathogenic genetic variants in the *PMM2* locus, from independent kindreds, extends the previously reported spectrum of PMM2-related HI/ARPKD disease. It identifies *PMM2* as a potential novel Mendelian association of early-onset IBD (age of onset 0, 6, and 10 years). We currently estimate a low penetrance of IBD of 10% (95% confidence intervals 3.5–25.6%) based on 30 patients in the literature (Cabezas et al. 2017; Moreno Macian et al. 2020; Prasher et al. 2020; Dorval et al. 2021), and 3 patients with IBD (described here—there have been no prior reports of IBD in this patient group).

The distinctive gastric manifestations, particularly prominent in P1 and P2, have not been previously described in the context of early-onset/monogenic IBD to our knowledge. Hyperplastic polyps are a common type of gastric polyp identified in adults, arising secondary to non-specific but significant gastric inflammation, especially due to *Helicobacter pylori*. They are extremely rare in children. The patients reported here never had *H. pylori* identified, never had prominent upper GI symptoms, and histopathologic inflammation in the stomach was always fairly mild (Kovari et al. 2021; Ouyang et al. 2021). Macroscopic and histopathologic findings were not in keeping with any of the juvenile polyposis syndromes or Menetrier’s disease. We consider that the presence of such an unusual gastric pathology (for their age) in a group of patients with the same genetic background lends credence to the concept of a genuine association between PMM2-HIPKD and gastrointestinal pathology as opposed to coincidence. In terms of management, malignant transformation of hyperplastic polyps is rare, but surveillance is recommended (Banks et al. 2019). For P1, at least, the appearances have remained stable over time, and it is notable that both P1 and P2 had macroscopic gastric pathology documented very early in life, and have, therefore, possibly been living with foveolar hyperplasia for many years. In the absence of symptoms or concerns for dysplasia, we have never attempted polypectomy.

**Fig. 2:**
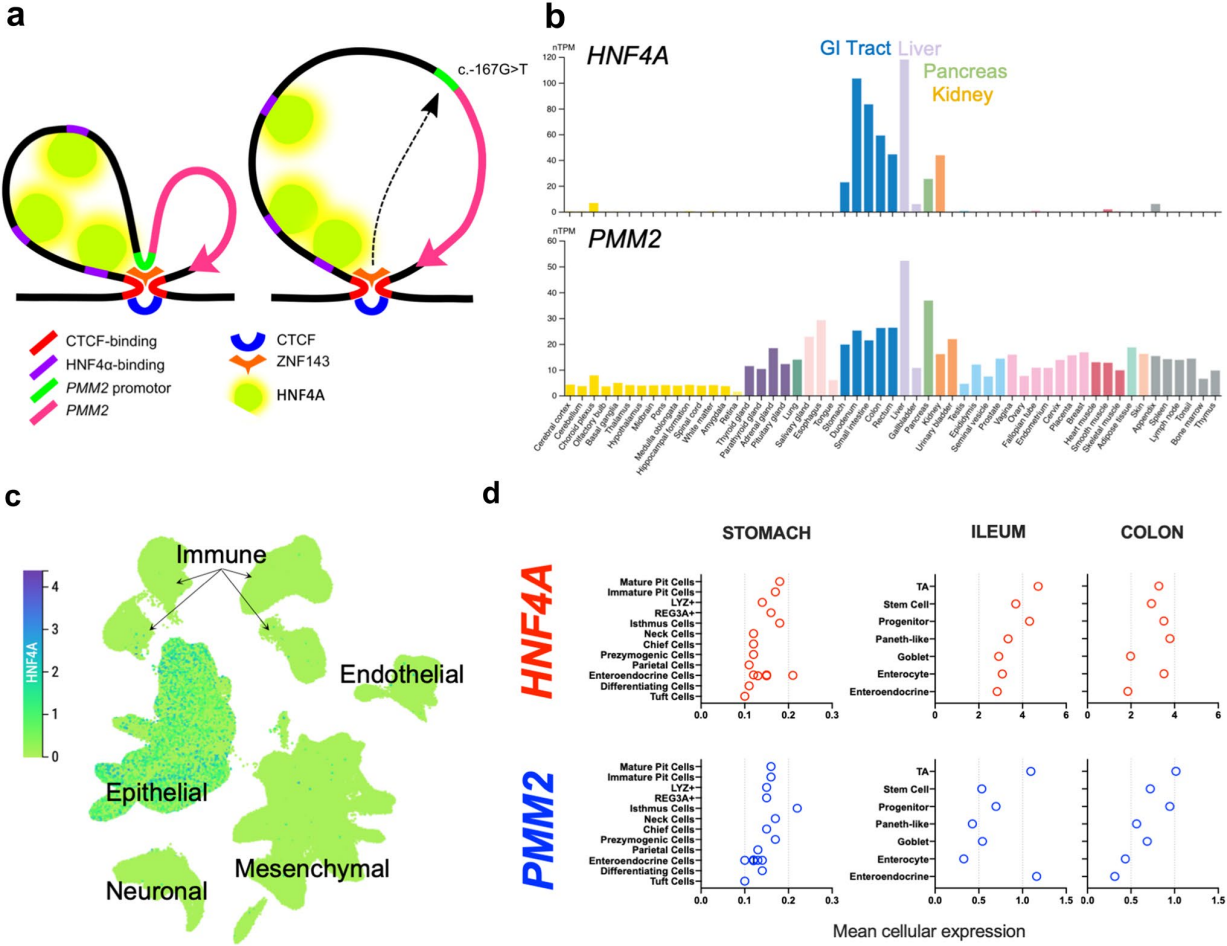
**a** Simplified cartoon illustrating proposed mechanism of organ/tissue specificity. ZNF143 binds the WT promotor (left) and CTCF-binding sites, altering the 3-dimensional structure of the CFTF delimited chromatin loop and bringing the PMM2 promotor into proximity with HNF4A binding sites. In cells where HNF4A is expressed, it is thereby able to interact with the PMM2 promotor and function as a cis-acting regulatory element. The c.-167G > T mutant promotor (right) has reduced affinity for ZNF143, disrupting the approximation of promotor and HNF4A binding sites and reducing the HNF4A-dependent transcriptional regulation (adapted from Rubio Cabezas et al. 2017). **b** Comparison of tissue level expression of *HNF4A* (upper bar chart), and *PMM2* (lower), (images available from The Human Protein Atlas v21.1 https://www.proteinatlas.org/ENSG00000140650-PMM2/tissue, https://www.proteinatlas.org/ENSG00000101076-HNF4A/tissue) (Uhlen et al. 2015): PMM2 is broadly expressed across tissues, whereas the tissue-specific expression of *HNF4A* closely matches the disease manifestations of patients with PMM2-HIPKD. **c** Single cell transcriptomic data from the Gut Cell Survey (www.gutcellatlas.org) (Elmentaite et al. 2021) illustrates that intestinal *HNF4A* expression is restricted to the epithelium (image from https://www.gutcellatlas.org/spacetime/full/). **d** Single cell transcriptomic data from human gastric epithelium (Busslinger et al. 2021) identifies isthmus cells as having the highest conjoint *HNF4A*/*PMM2* expression. In ileum and colon, *HNF4A* and *PMM2* are broadly expressed across cell types (Wang et al. 2020). Similar to the stomach, expression by stem- and progenitor cells is prominent

The proposed association between PMM2-HIPKD and intestinal inflammation is puzzling in view of the fact that comparatively large, longitudinal cohort studies have failed to identify any equivalent association with PMM2-CDG (Schiff et al. 2017; Altassan et al. 2019). However, this is also the case with the other key manifestations of hyperinsulinism and cystic kidney disease, both of which are ubiquitous in currently described PMM2-HIPKD cases but rare in PMM2-CGD (1%, and 2% of cases, respectively) (Altassan et al. 2018; Moravej et al. 2020). We have hypothesised that the distinctive pattern of organ involvement in HIPKD may be a consequence of the promotor variant interrupting an interaction with tissue-specific *cis*-acting regulatory elements (Fig. 2a). We have experimentally demonstrated that the c.-167G > T variant impacts ZNF143 binding, and suggested this may lead to destabilisation of a chromatin loop which, in the wild type, may bring the promotor into physical proximity with regulatory elements. The presence of multiple potential binding sites for HNF4A in the loop, along with the fact that HNF4A tissue-specific expression mirrors the pathology in HIPKD, led us to speculate that this transcription factor may underlie the organ specificity (Cabezas et al. 2017). The extension of HIPKD’s spectrum of disease to include the GI tract is consistent with this hypothesis since HNF4A expression seems to reflect all the organ pathology seen in PMM2-related disease, i.e. kidney (polycystic kidney disease), pancreatic (hyperinsulinaemic hypoglycaemia), liver (hepatic cysts), gastric (foveolar hyperplasia), and intestinal tissue (inflammatory bowel disease) (Fig. 2b) (Uhlen et al. 2015). However, even in HNF4A-expressing cells, the mechanism by which this transcription factor interaction impacts on PMM2 expression/function such that patients with PMM2-HIPKD experience a materially greater impact than those with PMM2-CGD is unclear. It is notable that in all three patients, the c.-167G > T promotor variant is *in trans* with the c.422G > A p.Arg141His variant, which has been shown to have the most substantial impact on PMM2 enzymatic activity—effectively null—and is never found in homozygosity (presumed embryologically lethal) (Yuste-Checa et al. 2015; Matthijs et al. 1998). It is plausible that in cells where HNF4A is important for the regulation of *PMM2* transcription, the PMM2-HIPKD combination of ‘blocked’ transcription of a normal variant (c.-167G > T) plus ‘null’ (p.Arg141His) might be associated with more-reduced cell type-specific transcription than that occurring in PMM2-CDG where there is typically ‘null’ (e.g. p.Arg141His) alongside a complementary allele that has less-severely reduced enzymatic activity and retains the capacity for HNF4A transcriptional control. Against this, our previous in vitro work with renal and pancreatic cell lines, and nephrectomy tissue from an affected patient, identified only a partial reduction in transcription with the promotor variant, and does not conclusively support a hypothesis that PMM2 enzyme activity is lower in HNF4A-expressing cells of PMM2-HIPKD patients versus those with PMM2-CDG—especially those harbouring more damaging combinations of variants. We can speculate that our prior in vitro work may not have captured the full range of cell type-specific effects of the promotor variant, and certain cell types in the gastrointestinal tract might be particularly severely affected. This is a testable hypothesis and should be addressed experimentally. Furthermore, HNF4A expression levels are dynamic and context specific, with a circadian periodicity (Qu et al. 2018), and impacted by diverse dietary and microbial cues (Lickwar et al. 2022). We speculate that this might engender a dynamic regulation of N-glycosylation that is somehow important for mucosal immune homeostasis.

We have considered whether the association between HIPKD and intestinal inflammation might be explained by some other common confounding exposure, for example medications for renal disease/hyperinsulinism, but have not identified any other likely candidates. Although P2 was immunosuppressed at point of development of IBD following his renal transplant, the de novo development of IBD in renal transplant recipients is extremely rare (Gioco et al. 2020).

The immunohistochemical staining undertaken reveals a normal distribution of HNF4A protein expression in the patients, with the expected restriction to the epithelial compartment. PMM2 is more widely expressed, but also most prominent in the epithelium. One tested patient had low PMM2 protein expression levels, whereas the other had levels comparable to control tissue. Unfortunately, we do not expect the available anti-PMM2 antibodies to distinguish between protein derived from non-mutated *PMM2* versus *PMM2* with a missense disease-causing variant (e.g. p.Arg141His). Therefore, although the protein is present, we expect the enzymatic activity to be low as it will predominantly reflect the p.Arg141His allele. There is some evidence that mutant protein is less stable than WT and this could contribute to the reduced levels seen in P1, but their discrepancy with P2 is not explained and we hesitate to draw any firm conclusions from such a small sample size (Yuste-Checa et al. 2015). To date, we have been unable to access biopsy tissue for further study (e.g. transcriptomic analysis, tissue-specific glycosylation), but suggest this could be a productive area for future clinical and experimental research.

The epithelial restriction of HNF4A we have demonstrated in the GI tract is in keeping with published data that indicate it is expressed across diverse intestinal epithelial cell subtypes (Fig. 2c, d) (Elmentaite et al. 2021; Wang et al. 2020). The N-glycosylation pathway, in which PMM2 has an essential role, is particularly important in facilitating release of proteins from the endoplasmic reticulum for extracellular secretion (Medus et al. 2017), and several specialised intestinal epithelial cells have functional roles that depend on protein secretion. Blocking N-glycosylation results in reduced MUC2 secretion and increased ER (endoplasmic reticulum) stress in goblet cells (Asker et al. 1998; Tawiah et al. 2018). Goblet cell ER stress has been implicated in the development of intestinal inflammation both in animal models and a recently described monogenic association of IBD involving *AGR2* (Al-Shaibi et al. 2021; Adolph et al. 2013). However, there is no goblet cell depletion evident in our patients, and goblet cell expression of *HNF4A* is notably low compared to other epithelial cells (Fig. 2d). In the stomach, reanalysis of existing single cell data identifies proliferative isthmus cells as the major cell type with the highest conjoint expression of *HNF4A* and *PMM2* (Fig. 2d) (Busslinger et al. 2021). In mice, targeted deletion of *Hnf4a* in the stomach is associated with enhanced isthmus cell proliferation and increased gastric unit length (Moore et al. 2016), and directed expression of the *Kras* oncogene in this lineage resulted in foveolar hyperplasia (Kinoshita et al. 2019). We, therefore, propose that the development of gastric antral foveolar hyperplasia in HIPKD reflects an epithelial-intrinsic dysregulation of isthmus cell proliferation.

Monogenic IBD can cause a severe and treatment resistant disease course, but PMM2-related IBD appears to be relatively mild form of IBD (best classified as IBD-unclassified (IBDU)) and responds to standard treatments (azathioprine, sulfasalazine, infliximab). None of these patients were considered for therapy escalation such as haematopoietic stem cell transplantation, and the gene expression data and the current model of pathogenicity suggest that would not be curative.

In summary, PMM2-HIPKD, arising consequent to variants in PMM2, is associated with early-onset inflammatory bowel disease and distinctive gastric pathology. With relatively low penetrance, a small number of patients, and no definitive explanatory mechanism we leave open the possibility of a chance association. Based on gene expression data, we propose that PMM2-HIPKD-IBD is taxonomically best categorised as an epithelial-intrinsic defect pending further functional characterisation (Bolton et al. 2022).

## Supplementary Information

Below is the link to the electronic supplementary material.

Supplementary Supplementary Figure 1. a: Representative duodenal pathology from P1’s first diagnostic endoscopy showing villous blunting, crypt hyperplasia, and a chronic inflammatory infiltrate in the lamina propria. b, c: Colonic pathology from P1’s diagnostic endoscopy showing severe inflammation, architectural distortion, ulceration with a mixed acute/chronic inflammatory infiltrate, and (c [detail of b]) cryptitis. d: Representative colonic pathology form P2’s diagnostic endoscopy showing moderate chronic active pancolitis file1 (TIFF 4263 KB)

Supplementary Supplementary Figure 2. Immunohistochemistry showing protein expression for HNF4A (a-c, g-i, m-o) and PMM2 (d-f, j-l, p-r) in gastric antrum, duodenum, and colon, respectively. Representative sections from an unaffected paediatric control (column 1), Patient 1 (column 2), and Patient 2 (column 3) are shown. Sections of formalin fixed paraffin embedded tissue were cut at 3μm thickness. Staining was performed on the Leica Bond-Max automated platform with Bond Polymer Refine Detection Kit with DAB Enhancer, Heat Induced Epitope Retrieval with Bond Epitope Retrieval Solution 2 (EDTA based) for 30 minutes, and antibody incubation for 30minutes. Antibodies were anti-PMM2 #HPA063649 (Atlas Antibodies) diluted 1:50 in BondTM Primary Antibody Diluent #AR9352; anti-HNF4A #HPA004712 (Atlas Antibodies) diluted 1:50 in BondTM Primary Antibody Diluent #AR9352 file2 (TIF 104918 KB)

## Data Availability

The datasets generated during the current study are available from the corresponding author on reasonable request.
