# Supplementary figures and images for: Phosphomannomutase 2 (PMM2) variants leading to hyperinsulinism-polycystic kidney disease are associated with early-onset inflammatory bowel disease and gastric antral foveolar hyperplasia
Source: Hum Genet. 2023 Feb 11;142(5):697–704. doi: 10.1007/s00439-023-02523-7 (PMC10181953; doi:10.1007/s00439-023-02523-7)

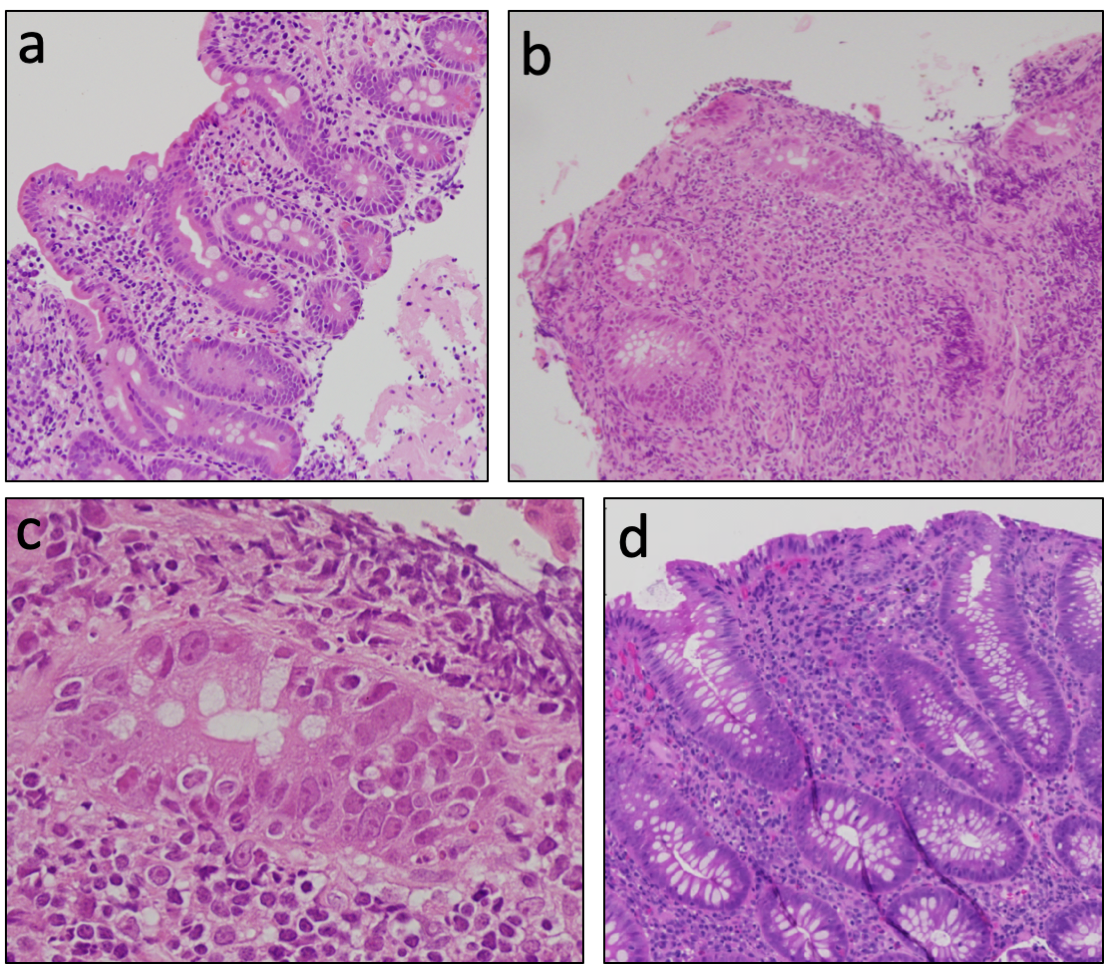

Supplement: Supplementary file 1 — Supplementary Supplementary Figure 1. a: Representative duodenal pathology from P1’s first diagnostic endoscopy showing villous blunting, crypt hyperplasia, and a chronic inflammatory infiltrate in the lamina propria. b, c: Colonic pathology from P1’s diagnostic endoscopy showing severe inflammation, architectural distortion, ulceration with a mixed acute/chronic inflammatory infiltrate, and (c [detail of b]) cryptitis. d: Representative colonic pathology form P2’s diagnostic endoscopy showing moderate chronic active pancolitis file1 (TIFF 4263 KB) [file 439_2023_2523_MOESM1_ESM.tiff]
